# Immortalized mammosphere-derived epithelial cells retain a bioactive secretome with antimicrobial, regenerative, and immunomodulatory properties

**DOI:** 10.1186/s13287-024-04019-1

**Published:** 2024-11-14

**Authors:** Nikola Danev, Julia M. Poggi, Emilie A. Dewever, Arianna P. Bartlett, Leane Oliveira, Lucas Huntimer, Rebecca M. Harman, Gerlinde R. Van de Walle

**Affiliations:** 1 Baker Institute for Animal Health, College of Veterinary Medicine, Cornell University, 235 Hungerford Hill Road, Ithaca, NY 14853 USA; 2 Elanco Animal Health, 2500 Innovation Way, Indianapolis, IN 46241 USA; 3 Department of Veterinary Pathobiology, Royal (Dick) School of Veterinary Studies and Roslin Institute, University of Edinburgh, Easter Bush, Roslin, Midlothian UK

**Keywords:** Mammary epithelial cells, Secretome, Immortalization, Immunomodulation, Antimicrobial effects, Fibroblast migration, Angiogenesis

## Abstract

**Background:**

The secretome of primary bovine mammosphere-derived epithelial cells (MDECs) has been shown to exert antimicrobial, regenerative, and immunomodulatory properties in vitro, which warrants its study as a potential biologic treatment with the potential to be translated to human medicine. Currently, the use of the MDEC secretome as a therapy is constrained by the limited life span of primary cell cultures and the decrease of secretome potency over cell passages.

**Methods:**

To address these limitations, early-passage bovine MDECs were immortalized using hTERT, a human telomerase reverse transcriptase. The primary and immortal MDECs were compared morphologically, transcriptomically, and phenotypically. The functional properties and proteomic profiles of the secretome of both cell lines were evaluated and compared. All experiments were performed with both low and high passage cell cultures.

**Results:**

We confirmed through in vitro experiments that the secretome of immortalized MDECs, unlike that of primary cells, maintained antimicrobial and pro-migratory properties over passages, while pro-angiogenic effects of the secretome from both primary and immortalized MDECs were lost when the cells reached high passage. The secretome from primary and immortalized MDECs, at low and high passages exerted immunomodulatory effects on neutrophils in vitro.

**Conclusions:**

High passage immortalized MDECs retain a bioactive secretome with antimicrobial, regenerative, and immunomodulatory properties, suggesting they may serve as a consistent cell source for therapeutic use.

**Supplementary Information:**

The online version contains supplementary material available at 10.1186/s13287-024-04019-1.

## Background

Bacterial infections cause a significant burden on society. In 2019, bacterial pathogens killed an estimated 7.7 million people, making bacteria the second leading cause of death worldwide [1]. Every human organ is susceptible to infection, and some bacteria, such as *Staphylococcus (S.) aureus*, may infect almost any site in the body and spread systemically through the bloodstream [2]. The pathogenesis of bacterial infections often results from tissue damage caused by host immune responses rather than direct bacterial activity [3]. Some bacterial infections may not cause extensive tissue damage but are problematic because the pathogens evade the host immune response, allowing the grow and proliferation inside of host cells, and thus, disrupting homeostasis [4]. Antibiotics, the most common treatment for bacterial infections, have various mechanisms of action that either inhibit or kill bacteria [5]. However, despite the recognized effectiveness of antibiotics in combatting infections, they are not designed to address the negative consequences of tissue damage due to host immune responses or bacterial immune evasion strategies.

A therapeutic alternative to antibiotics is the use of biologics. Biologics are produced in microorganisms or mammalian cells, as opposed to being generated by chemical manufacturing processes [6]. One type of biologic is the cell secretome, which contains all bioactive factors secreted by the cells. The secretome from cultured cells can be collected as conditioned medium (CM) [7]. The secretome from stem and progenitor cells from multiple species has been found to improve tissue damage and reduce bacterial immune evasion [8–18]. For example, the stem cell secretome has been shown to enhance fibroblast migration that supports wound healing and tissue remodeling, processes that are delayed by bacterial infections [19–21]. The stem cell secretome can increase angiogenesis, which may promote the revascularization of damaged tissues, reducing the ischemia which can occur as the result of bacterial infection [22, 23]. Finally, by modulating the immune response, the stem cell secretome may reduce host immune response-derived tissue damage and bacterial immune evasion [16, 24–29].

Previous work by our group identified stem cell secreted factors with the potential to decrease bacterial growth and viability and promote the regeneration of tissue. Specifically, work in the bovine model has shown that the secretome of adult mammary stem/progenitor cells confers antimicrobial, regenerative, and immunomodulatory properties in vitro [16, 17, 30].

While biologics provide alternatives or adjuncts to conventional antibiotic approaches, the collection of the secretome from primary cell lines can be challenging at scale and over long periods of time. The activity of secretome-derived biologic products is influenced by the characteristics of individual cells lines, as well as their growth conditions, requiring significant efforts to ensure batch consistency and reproducibility [6, 13]. One major obstacle to batch consistency and scalability is the loss of proliferative capacity of primary cell lines over multiple passages [31]. After successive subculturing, we found that certain primary mammosphere-derived epithelial cell (MDEC) cultures exhibit a decreased ability to proliferate, lose their self-regenerative phenotypes, and become less viable [32, 33].

The goal of this study was to immortalize an early-passage primary bovine MDEC line by transfecting the cells with the immortalization gene *hEST2* (human *EST2*)*,* also known as *hTERT* (human telomerase reverse transcriptase) [34, 35]. Telomeres play a critical role in maintaining the termini of chromosomes, protecting the chromosomes from damage [36]. A loss of telomeres leads to the cessation in replicative processes, limiting a cell’s ability to divide [36]. Telomerase, ubiquitously coded in eukaryote DNA, maintains and extends telomeres, however, the expression of telomerase is strongly repressed in most somatic tissues [37]. By integrating *hTERT*, the catalytic subunit of the telomerase enzyme, into a cell genome, telomeres are maintained or even extended [38]. This overrides the replicative limit of cells, theoretically allowing them to be passaged indefinitely in culture. By introducing *hTERT* to primary MDECs, we found that MDECs can be immortalized, and that the secretome of high passage immortalized MDECs has antimicrobial, pro-migratory, and immunomodulatory effects on target cells. Collectively, we propose immortalized bovine MDECs as a viable source of secreted bioactive molecules to treat bacterial infections and to inform future secretome-based biologic approaches in translational medicine.

## Methods

### Bovine stem/progenitor cell lines

Mammary gland tissues were collected and cryopreserved for a previous study and mammosphere-derived epithelial cell (MDEC) lines were generated exactly as described previously [17]. Briefly, cryopreserved samples were thawed at 37 °C and dissociated using 0.25% trypsin-ethylenediaminetetraacetic acid (EDTA) (Corning, Corning, NY), dispase (Sigma-Aldrich, St. Louis, MO) and DNAse-1 (Worthington, Lakewood, NJ) and triturated. To obtain single cells, digested tissue material was filtered through 100 µm and then 40 µm cell strainers and then seeded at 1 × 10^6^ cells were per well of a 6-well tissue culture plate for 1 h (h), allowing adherent cells to attach. The supernatants, enriched for epithelial cells in suspension were transferred to new wells of a 6-well plate for another 1 h (h). The supernatants, further enriched for epithelial cells were transferred to a 6-well ultralow attachment plate to enrich for stem/progenitor cells through the formation of mammospheres. Approximately 14 days after plating, mammospheres were transferred to adherent plates and allowed to form 2 dimensional MDEC cultures, which were then used for downstream experiments. MDECs were cultured in epithelial stem cell (EpSC) medium, consisting of Dulbecco’s Modified Eagle Medium (DMEM) and Ham’s F12 (50/50) (both from Corning), supplemented with 10% FBS (R&D Systems, Minneapolis, MN), 2% B27 (Invitrogen, Waltham, MA), 10 ng/mL basic-fibroblast growth factor (bFGF) (BioVision, Milpitas, CA), 10 ng/mL epidermal growth factor (EGF) (Sigma, Darmstadt, Germany) and 2% Penicillin/Streptomycin (P/S) (Corning) [16, 17].

Bovine mesenchymal stem/stromal cells (MSCs) from bovine adipose tissue (AD), bone marrow (BM), and peripheral blood (PB), that have been published previously [15] and cryopreserved in liquid nitrogen, were expanded after thawing in MSC Expansion Medium, consisting of low glucose DMEM (Corning), with 30% FBS (R&D Systems), 2% P/S (Corning) and 2% L-glutamine (Corning).

### Immortalization and selection of MDECs

A Lonza Nucleofector II electroporation device (Basel, Switzerland) was tested, per manufacturer’s protocols, to determine if electroporation was an effective way to transfect MDECs with plasmid DNA. A pmaxGFP vector (included by manufacturer) was transfected into MDECs cultured in Amaxa Human Mammary Epithelial Cell medium (Lonza) using the “Y-001” instrument setting. Approximately 20% of cells were transfected under these conditions (Supplemental Fig. 1a). As the immortalization gene *hEST2* would be introduced in a plasmid containing a G418 resistance gene, a 3-(4,5-dimethylthiazol-2-yl)-2,5-diphenyltetrazolium bromide (MTT) assay (Thermo Fisher) was performed, according to manufacturer’s instructions, to determine the lethal concentration of the antibiotic G418 (Thermo Fisher) on MDECs (Supplemental Fig. 1b). Based on these data, a concentration of 700 µg/ml G418 was used for selection.

**Fig. 1:**
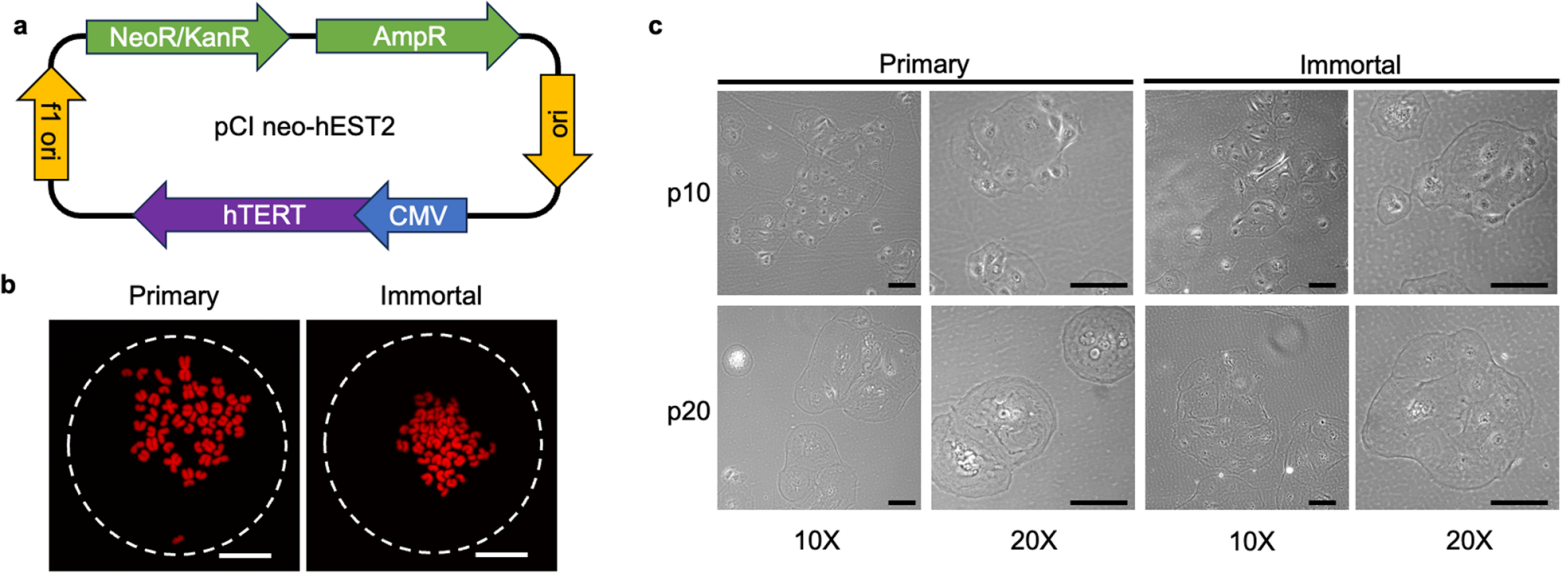
Immortalization of bovine mammosphere-derived epithelial cells (MDECs) does not alter genomic integrity or phenotype. **a** Graphical representation of the pCI neo-hEST2 plasmid used to transfect primary MDECs. The plasmid expresses hEST2 (hTERT) under a constitutive CMV promoter and can be selected using the AmpR gene in bacteria and the NeoR/KanR gene in mammalian models. **b** Representative images of chromosome spreads of the primary and immortalized MDECs at p10 indicating a consistent number of chromosomes in both cell lines. Scale bar = 25 μm. **c** Representative images of phase ring light microscopy of primary and immortalized MDECs adhering on a plastic plate at p10 and p20 at 10× and 20× magnification. Scale bars = 50 μm

After validation of the transfection technique and determination of the lethal dose of G418, a plasmid containing human *EST2* (*hTERT)* under a CMV promoter, as well as a G418 resistance gene (NeoR/KanR), was used to transfect MDECs (Fig. 1a). The plasmid, pCl neo-*hEST2*, was a gift from Bob Weinberg [39] (Addgene plasmid # 1781). To transfect MDECs with the pCl neo-*hEST2* plasmid, 2 × 10^5^ cells were centrifuged at 200 × g for 6 min at room temperature (RT). The pellet was then resuspended in 100 µL Nucleofector solution (Lonza) at RT and combined with 2 µg plasmid. The suspension was transferred to a transfection cuvette and placed in a Lonza Nucleofector for transfection. Immediately following transfection, cells were transferred to a pre-warmed well of a 6-well plate with EpSC medium. Selection for G418-resistant cells was initiated after 24 h by supplementing EpSC medium with 700 µg/mL G418. Cells were cultured in EpSC + G418 medium for 21 days until three distinct colonies developed. Single colonies were then isolated and allowed to expand separately in EpSC medium. One colony did not expand and was discarded from further experiments. Of the remaining two, one was selected for further analysis. A representative image of the post-selection immortalized MDECs showing typical MDEC morphology is shown in Supplemental Fig. 1c.

### Phase-ring light microscopy

Primary and immortalized MDECs on adherent culture plates were visualized on a CKX41 phase-ring inverted light microscope (Olympus, Waltham, MA) and imaged using an Infinity2-1R USB Microscopy Camera (Lumenera, Ottawa, ON).

### Chromosome spread assay

Primary and immortalized MDECs at passage (p)10 grown to 80% confluence in a T75 tissue culture flask were incubated with 0.2 µg/mL colcemid (Thermo Fisher) overnight to synchronize cells in metaphase. The following day, glass slides were pre-washed with a methanol/acetic acid (3:1) mixture, after which they were chilled for 1 h in ice cold water. Cells were washed with sterile Phosphate Buffered Saline (PBS) (Corning) and detached from flasks using 0.25% Trypsin–EDTA for 10 min. The trypsin reaction was inactivated with EpSC medium, and cells were pelleted at 300 × g for 5 min at RT. Cell pellets were resuspended dropwise in 1 mL 0.56% KCl, after which an additional 4 mL of KCl was dispensed using a serological pipette. Cells were incubated for 6 min at RT, pelleted by centrifugation at 200 × g for 5 min at RT, and resuspended in 1 mL of the methanol/acetic acid (3:1) mixture. After 5 min of incubation, cells were pelleted at 200 × g for 5 min, for a total of 3 times, and finally resuspended in 1 mL of the methanol/acetic acid (3:1) mixture. Slides were removed from ice cold water and edges were blotted to remove excess liquid. Cell suspensions were dropped in single droplets from 2 m height onto the slides. Slides were left to dry at RT and then stained with SYTOX Orange DNA stain (Thermo Fisher) at a 1:25,000 dilution in PBS. Coverslips were applied using aqueous mounting medium and slides were left to dry in the dark at RT. Chromosome spreads were imaged on a Fluoview FV300 confocal microscope (Olympus) and analyzed using ImageJ [40].

### Polymerase chain reaction (PCR) assays

PCR was performed, exactly as previously described [14]. For quantitative reverse transcriptase PCR (qRT-PCR), manufacturer’s instructions were followed to isolate RNA using the RNeasy Mini Plus kit (Qiagen, Hilden, Germany) and synthesize cDNA using the iScript Clear cDNA synthesis kit (BioRad, Hercules, CA). Primers were generated using Primer3 [41] with original sequence data from NCBI GenBank (Table 1). As previously outlined, primers were designed to span intronic regions when possible [16]. PowerTrack SYBR Green MasterMix (Thermo Fisher) was used for the PCR reaction, which was performed using a QuantStudio3 thermal cycler (Thermo Fisher). Data were analyzed and visualized using RStudio (Posit, Boston, MA) with the packages cowplot [42] and ggplot2 [43].

**Table 1:** Primers used for PCR and RT-qPCR

Target	Gene name	Forward (5′– > 3′)	Reverse (3′– > 5′)	Source
LYAR	Ly1 antibody reactive	CAGCAGTGAACCTGTTGGTAAG	ATTCTTCTTGGCCTCTGCTG	#
ERa	Estrogen receptor alpha	CGCAAGTGCTATGAAGTGG	CGCTTGTGCTTCAACATTC	#
DUSP6	Dual specificity phosphatase 6	CTACCTGGAAGGTGGTTTCAG	ATGTCCGAGGAGGAGTCG	#
CD168	Cluster of differentiation 168	TGAGGGGAAACTTGTTTCAATAG	CCACTTGATCTGATGCACAAC	#
CD49f	Cluster of differentiation 49f	CTGGAAAGGGATTGTTCGTG	TCATCATGGTCAGTCTCTCCAC	#
CD44	Cluster of differentiation 44	GACACGTACTGCTTCAATGCCTC	GTTAGTTCTGTATTCGCCTTTCTTGGT	#
CK18	Cytokeratin 18	TCTGTGGAGAGTGACATACACG	CCCTTTACTTCCTCCTCATGG	#
CK14	Cytokeratin 14	GAGGAACAAGATCCTCACAGC	TTCAGCTCCGTCTCGTACTTG	#
VEGF	Vascular endothelial growth factor	CCCACGAAGTGGTGAAGTTC	CCACCAGGGTCTCGATGG	[44]
FGF2	Fibroblast growth factor 2	CGAGAAGAGCGACCCACAC	GCCCAGTTCGTTTCAGTGC	[45]
TBK1	TANK-binding kinase 1	CACCAAGCTGTTGAGACTTTCC	AGTGCCTTCTTGATGGGTCC	[46]
GAPDH	Glyceraldehyde-3-phosphate dehydrogenase	CACAGTCAAGGCAGAGAACG	TACTCAGCACCAGCATCACC	#
NeoR/KanR	Neomycin/Kanamycin resistance	GATGGATTGCACGCAGGTTC	AAAAGCGGCCATTTTTCCACC	#

^#^Indicates in-house made primers

### Immunofluorescent antibody binding (IF)

IF was performed, as previously described [16]. Briefly, MDECs were detached from culture wells and 1,000 cells were plated on 12 mm glass coverslips seated in 24-well plate wells. Cells were fixed with 4% paraformaldehyde (PFA; Thermo Fisher) and where needed, permeabilized with 0.1% Triton (Alfa Aesar, Ward Hill, MA) in PBS for intracellular antibody binding. Coverslips were incubated in primary antibodies at 4°C overnight, and then in secondary antibodies for 1 h at RT (Table 2). SYTOX Orange was used as a counterstain for DNA. Coverslips were mounted on slides using DAKO aqueous mounting medium (Agilent Technologies, Santa Clara, CA) and cells were imaged on a Fluoview FV300 confocal microscope. Image processing and analysis was performed using ImageJ [40].

**Table 2:** Antibodies used for immunofluorescent imaging

Target	Host	Clone	Dilution	Manufacturer	Identifier
Vimentin	Mouse	RV202	1:100	Abcam	Cat# ab8978
Alpha smooth muscle actin	Mouse	1A4 (conjugated to AF594)	1:100	Abcam	Cat# ab202368
Cytokeratin 14	Mouse	LL002	1:100	Abcam	Cat# ab7800
Estrogen receptor alpha	Mouse	T111.5D11	1:100	Abcam	Cat# ab16460
Cytokeratin 18	Mouse	C-04	1:100	Abcam	Cat# ab668
Mouse IgG (H + L)	Goat	N/A (conjugated to AF488)	1:500	Jackson	Cat# 115-545-166

### Population doubling time (PDT) assay

Primary and immortalized MDECs at p9 were seeded at 1 × 10^6^ cells per T75 flask and grown in EpSC medium until 80% confluence was reached. Cells were then split into three technical replicates by seeding at 2 × 10^5^ cells in 3 T25 flasks in EpSC medium. Cells were observed daily for growth and upon reaching 80% confluence, were detached using 0.25% Trypsin–EDTA and counted. Cells were re-plated in T25 flasks at a starting concentration of 2 × 10^5^ and this was repeated for 10 passages, from p10 to p20, for both primary and immortalized MDECs, in triplicate. PDT was calculated based on cell counts, using an online calculator [47].

### Mammosphere and colony forming assays

Mammosphere-forming capacity (MFC) assays were conducted, as previously described [16, 32, 48]. Briefly, MDECs were plated in an ultra-low attachment 96-well plate at the following densities: 2, 5, 10, 25, 50, and 100 cells per well in 8 technical replicates and cultured for 10 days to allow mammospheres to form. Each well was imaged and mammosphere diameters were measured, with structures ≥ 50 µm being considered positive. Mammosphere frequency was determined using online software for extreme limiting dilution analysis [48].

Colony-forming unit (CFU) assays were performed following a previously established protocol [32]. MDECs were plated in a T75 flask at a concentration of 375 cells/flask in 10 mL EpSC medium and incubated for 10 days. Cells were fixed and stained with 0.1% crystal violet in 20% EtOH for 10 min, washed with DI water, and left overnight to dry. Colonies were counted manually under a light microscope.

### Collection of conditioned medium (CM)

CM was collected, as described previously [16, 17, 49, 50]. Briefly, cells were cultured in their corresponding antibiotic-free culture medium for at least 2 subcultures at a concentration of 1 × 10^6^ per T75 flask. Cells were washed twice with PBS and cultured in 6mL DMEM for 24 h for all assays, except for those involving neutrophils and mass spectrometry. For the neutrophils assays, CM was generated in RPMI (Corning) + 2% FBS, and in phenol red-free, FBS-free DMEM (Thermo Fisher) for the mass spectrometric analyses. CM was collected and centrifuged at 300 × g for 10 min at RT, decanted into a new tube, and centrifuged again to remove cellular debris.

### Neutrophil isolation

Bovine neutrophils were isolated, exactly as previously described [29]. Briefly, a licensed veterinarian collected 40 mL of blood from the coccygeal vein of each of three female, lactating, Holstein-Frisian cows into evacuated tubes containing ethylenediaminetetraacetic acid (EDTA). Blood collection was covered by Cornell University IACUC #2014–0038. Blood was diluted with 1:1 PBS, layered on Histopaque (Sigma Aldrich) and centrifuged at 1000 × g for 30 min at 18 °C with no brake. Interphases, Histopaque, and plasma were aspirated and discarded, leaving a pellet consisting primarily of neutrophils red blood cells. 0.2% NaCl was added to the pellet for 10 s to lyse red blood cells. Immediately following this step, an equal volume of 1.6% NaCl was added, and tubes were filled with PBS. Cells were centrifuged at 250 × g for 10 min at 4 °C. Lysis was repeated, after which cells were washed once by centrifugation with PBS and resuspended in RPMI medium (Corning) + 1% HEPES (Thermo Fisher). Viability was assessed using Trypan blue and percentage of neutrophils was calculated by visualization. Average viability was 98.3%, with 99.3% of all cells being neutrophils.

### Functional secretome assays

All assays were performed with CM from low passage primary, low passage immortalized, high passage primary and high passage immortalized MDECs. CM was also collected from bovine adipose tissue-derived (AD), bone marrow-derived (BM) and peripheral-blood derived (PB) mesenchymal stem/stromal cells (MSCs) and used in antimicrobial assays.

Antimicrobial assays were conducted with minor modifications from previously published protocols [16, 17]. A single clonal population of *Methicilin-resistant Staphylococcus aureus* (MRSA) was inoculated into Luria–Bertani (LB) broth and grown on an orbital shaker overnight at 37 °C. Bacteria were diluted to a concentration of 100 colony forming units (CFU)/µL and 750 µL of CM was inoculated with 250 µL of diluted bacteria. The inoculum was aliquoted into four wells of a 96-well plate at 200 µL/well for a concentration of 5000 CFU/well. Four technical replicates of DMEM and DMEM supplemented with 2X penicillin/streptomycin (Corning) were included as negative and positive controls, respectively. Plates were covered and incubated for 8 h at 37°C. Absorbance was measured at 600 nm at the 8-h timepoint.

Fibroblast migration assays were carried out, as previously described [16, 51]. Bovine mammary fibroblasts (previously isolated from the same donor cow [17]) were grown to confluence in a 6-well plate and mitomycin C (Sigma Aldrich) was added at a concentration of 20 µg/mL to inhibit mitosis. The fibroblast monolayer was disrupted by a scratch down the middle of the plate using a P1000 pipette tip. Cell debris was washed off and the remaining fibroblasts were incubated with either 2mL CM or 2mL DMEM + 1% FBS, both with mitomycin C at a concentration of 20 µg/m. Images of the scratch were immediately taken at two locations, and then the same locations were imaged 24 and 48 h later. ImageJ was used to calculate the percentage of area without cells (scratch percentage) at each timepoint.

Angiogenesis assays were conducted using an Abcam angiogenesis kit, per manufacturer’s instructions and as done previously [16, 50]. Briefly, bovine lung microvascular endothelial cells (BLMVEC) (gift from Dr. Theresa Curtis, State University of New York at Cortland) were plated on extracellular matrix in a 96-well plate and incubated with either 200 μL CM or DMEM + 1% FBS for 8 h at 37°C. BLMVEC were imaged using a phase-ring microscope. Three phase-ring images from each well were analyzed using the Angiogenesis Analyzer for ImageJ [52].

For neutrophil chemotaxis assays, 750 µL of each of the following conditions was added to 3 wells of a 24-well tissue culture plate: (i) RMPI + 2% FBS (negative control), (ii) RPMI + 2% FBS + Interleukin 8 (IL8) (positive control), and (iii) CM. Transwell inserts with 3.0 µm pores (Corning) were placed in each well and 50,000 neutrophils were seeded on the top surface of each insert. Neutrophils collected from 3 cows were tested against each treatment and the plates were incubated for 1 h at 37°C before inserts were removed. Plates were centrifuged at 300 × g for 5 min at RT to collect migrated cells at the bottoms of the wells, and medium was carefully removed. Neutrophils were fixed, stained with crystal violet, and imaged on a light microscope (Olympus). Numbers of migrated cells were counted and recorded in a blinded manner.

For neutrophil reactive oxygen species (ROS) assays, 1 × 10^6^ neutrophils in 100 µL of RPMI isolated from 3 different cows were aliquoted across 4 mL flow cytometry tubes. 600 µL of RPMI or CM was added, and tubes were incubated for 1 h at 37°C with 5% CO_2_. Cells were washed with RPMI + 2% FBS by centrifugation at 300 × g for 5 min at RT. The fluorescent dye H2DCFDA (Sigma Aldrich) diluted in RPMI + 10 mM HEPES was added for a 7.5 µM concentration and cells were incubated for 15 min at 37°C with 5% CO_2_. Cells were washed again by centrifugation and 25 ng/ml phorbol myristate acetate (PMA, Sigma Aldrich) in RPMI + 10 mM HEPES was added to appropriate tubes. Cells were incubated for 15 min at 37°C with 5% CO_2_, after which tubes were put on ice to stop the reaction before analysis on a BD LSRFortessa X-20 flow cytometer with FACSDiva software (BD Biosciences, Franklin Lakes, NJ). FlowJo software (BD Biosciences) was used to calculate mean fluorescent intensity (MFI) of samples and controls without H2DCFDA.

### Mass spectrometry and downstream analysis

Methods described previously were used for mass spectrometry and analysis [53, 54]. A detailed step-by-step protocol of the mass spectrometric assay is included in Supplement 1.

For downstream analysis, readouts for both fractions of CM of each sample were combined and duplicates were removed in Microsoft Excel. Data was imported into RStudio and analyzed using ggplot2 [42], ggvenn [55], dplyr [56], UpSetR [57], org.Bt.eg.db [58] and gprofiler2 [59]. Accession codes for each identified protein were extracted and the ‘ggvenn’ and ‘upset’ functions were used to generate a Venn diagram and UpSet plot of overlapping and unique proteins identified by condition. Then, the ‘gost’ function was applied to find GO term enrichment from the “GO:BP” source. Highlighted nodes were selected and “publish_gostplot” was used to generate a Manhattan plot of enriched GO terms in each CM sample.

### Statistical analysis

All statistical analyses were done in Prism 10 (GraphPad Software, La Jolla, CA). Data was collected in triplicate for qPCR assays and analyzed by one-way analysis of variance (ANOVA). PDT assays were performed in triplicate between p10 and p20. Data points were plotted, and a best-fit line and slope were extrapolated. Colony-forming and mammosphere assays were conducted in triplicate and Tukey’s ANOVA was performed. Antimicrobial assays were performed in triplicate, with four technical replicates averaged per assay, and a two-way ANOVA was performed. Fibroblast migration assays were performed in triplicate and simple linear regression was performed to identify best-fit values for slope, standard error and 95% confidence intervals. Angiogenesis data was collected from two assays, both with three replicates, and data were analyzed using a one-way ANOVA. All neutrophil assays were performed in triplicate using a two-way ANOVA.

## Results

### Immortalized bovine mammosphere-derived epithelial cells (MDECs) maintain morphology and marker expression profiles

Primary bovine MDECs were immortalized using a plasmid containing a copy of the human *EST2* (*hTERT*) gene under a constitutive CMV promoter (Fig. 1a) and insertion of the vector plasmid was verified by PCR amplification of the *NeoR/KanR* gene (Supplemental Fig. 1d). Immortalized MDECs were successfully propagated up to 38 subcultures whereas primary MDECs were only able to proliferate up to approximately 28 subcultures.

To confirm the chromosomal stability of both primary and immortalized MDECs, chromosome spreads were performed and a normal diploid phenotype of 60 chromosomes was observed in both cell lines (Fig. 1b). To verify gross morphological characteristics of both cell lines, phase-ring brightfield imaging was used. At p10, both primary and immortalized cells exhibited a characteristic cobblestone-like epithelial morphology, with cells localizing in patches. At p20, however, primary MDECs trended towards a larger and less uniform morphology compared to immortalized MDECs (Fig. 1c).

**Fig. 2:**
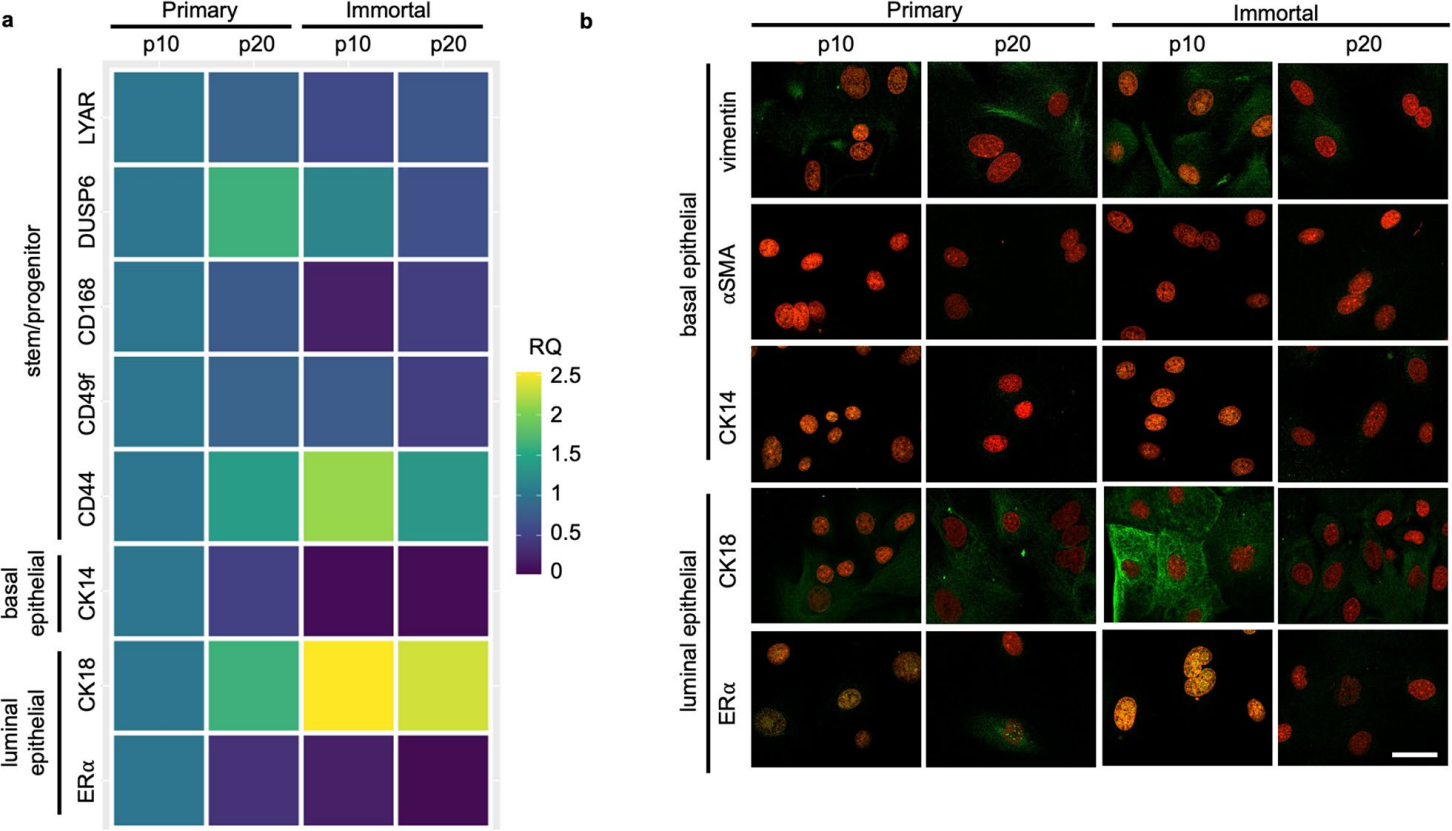
Mammosphere derived epithelial cell (MDEC) marker expression remains consistent in immortalized cells at transcriptomic and proteomic levels. **a** Results from qPCR analysis were plotted on a heatmap to show transcript expression in primary and immortalized lines at p10 and p20. Colors depict relative quantification (RQ) values where blue corresponds to low and yellow to high values. Stem and progenitor marker genes (*LYAR, DUSP6, CD168, CD49f* and *CD44)*, as well as mammary basal epithelial (*CK14*) and mammary luminal epithelial (*CK18* and *ERa*) are shown. **b** Representative images of accepted markers for bovine MDEC identification are compared across the four cell line conditions. Vimentin, aSMA and CK14 represent basal markers, and CK18 and ERα are luminal markers. Target proteins are labeled with green, nuclei are red. Scale bar = 50 μm

Each cell line was assessed at p10 and p20 for expression of commonly used markers to characterize bovine mammary stem/progenitor cell populations, at both the mRNA and protein levels. Transcripts for stem/progenitor markers *LYAR, DUSP6, CD168,* and *CD49f* [60–63] largely remained expressed consistently between p10 and p20 of the primary and immortalized cells, respectively, except for *DUSP6*, which was markedly increased between p10 and p20 in primary MDECs and decreased from immortalized p10 to p20 (Fig. 2a and Supplemental Fig. 2). In contrast, the stem/progenitor marker *CD44* [64] showed a greater variability in expression between primary and immortalized MDECs at p10, but not at p20 (Fig. 2a and Supplemental Fig. 2). Expression of *CK14*, a basal epithelial mammary cell marker [65], remained consistently expressed between p10 and p20 in the immortalized, but not the primary, population and compared to primary MDECs, *CK14* decreased in immortalized MDECs (Fig. 2a and Supplemental Fig. 2). Conversely, *CK18,* a luminal epithelial mammary cell marker [65], increased in immortalized cells relative to their primary counterparts (Fig. 2a and Supplemental Fig. 2). The other luminal marker assessed, *ERα* [17], trended downwards between p10 and p20 in primary as well as immortalized MDECs irrespective of passage, albeit this did not reach a statistical significance difference (Fig. 2a and Supplemental Fig. 2). Using immunofluorescent imaging to evaluate marker expression at a protein level showed consistent expression of the basal epithelial mammary cell marker vimentin, but not αSMA and CK14, and the luminal epithelial mammary cell markers CK18 and ERα [17] in both cell lines and across passages (Fig. 2b).

### Immortalized bovine MDECs maintain their population doubling time over multiple passages and show improved and consistent mammosphere-formation colony-forming capacity, respectively

**Fig. 3:**
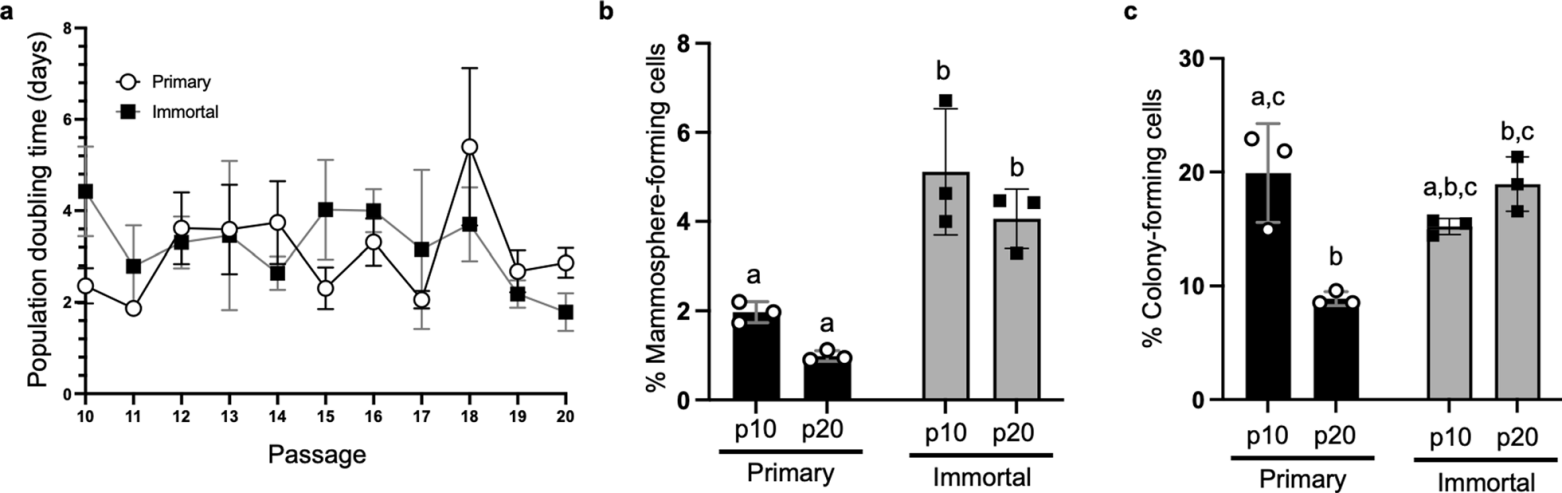
Mammosphere derived epithelial cell (MDEC) culture growth kinetics and cell composition are variably affected by immortalization and passage. **a** Population doubling time in days of primary and immortalized MDECs at each passage between p10 and p20. **b** Percentage of mammosphere forming cells in primary and immortalized p10 and p20 MDECs. **c** Percentage of colony forming cells in primary and immortalized p10 and p20 MDEC cultures. n = 3, *p* < 0.05. Different letters on graphs indicate statistically significant differences between groups

Proliferative capacity of primary cells in culture slows over multiple subcultures [66]. To assess this in primary and immortalized MDECs, population doubling time (PDT) was calculated in triplicate at every passage between p10 and p20. At p10, primary MDECs doubled at roughly twice the rate of the immortalized cells, however, immortalized MDECs proliferated faster at p20 when compared to their primary counterparts (Fig. 3a). Overall, PDT of primary MDECs remained relatively constant, with a slight upward trend, indicative of a sustained, albeit slightly slowing, growth rate (best-fit slope = 0.04), whereas the PDT of immortalized MDECs trended downwards, indicative of a sustained, slightly increasing growth rate (best-fit slope = − 0.13) (Fig. 3a).

Two common methods to assess stem and progenitor capacity of MDECs are mammosphere-forming capacity (MFC) and colony-forming unit (CFU) assays [32]. While neither primary nor immortalized MDECs had a significant change in MFC between p10 and p20, immortalized MDECs did have a significantly higher MFC at both p10 and p20 when compared to primary MDECs at the same passages (Fig. 3b). CFU assays showed a significant decrease in the CFU capacity of primary MDECs over 10 passages, whereas the CFU from immortalized MDECs did not change from p10 to p20 (Fig. 3c), highlighting the ability of immortalized MDECs to maintain their stem and progenitor capacities.

### The secretome from immortalized MDECs maintains antimicrobial, pro-migratory, and immunomodulatory properties, but loses angiogenic potential

The secretome from bovine adult mammary progenitor/stem cells, collected as conditioned medium (CM), has previously been shown to exhibit antimicrobial, regenerative (i.e. pro-migratory and angiogenic), and immunomodulatory properties in vitro [16, 17, 29]. To assess whether immortalization affects these characteristics of the MDEC secretome, we compared the effects of the CM from primary and immortalized MDECs at different passages on bacterial growth, fibroblast migration, endothelial cell morphology, and neutrophil function.

**Fig. 4:**
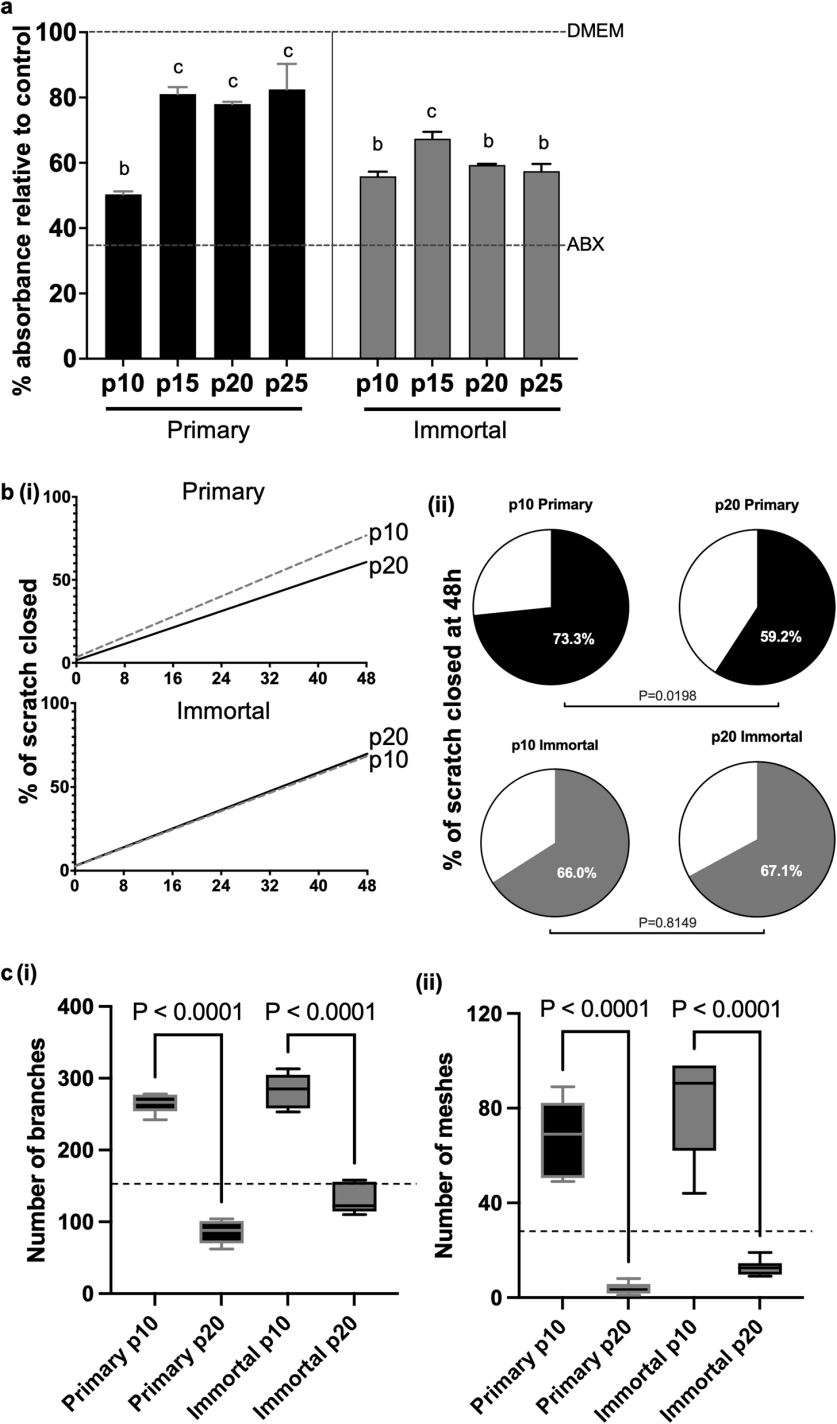
The antimicrobial and pro-migratory effects of immortalized, but not primary mammosphere derived epithelial cell (MDEC) conditioned medium (CM) are maintained over time in culture while angiogenic potential is lost in both primary and immortalized MDEC CM. **a** Relative absorbance of *MRSA* when co-cultured with CM from primary and immortalized MDECs at various passages. Upper dotted line represents the absorbance of *MRSA* cultured in unmodified DMEM (control medium), and the lower dotted line represents a positive control of medium with antibiotics. Comparisons were repeated over 4 passages and performed for each timepoint in four technical replicates. Different letters on graphs indicate statistically significant differences between groups. **b** (i) Percentage of scratch closure in a monolayer of bovine fibroblasts cultured in primary and immortalized MDEC CM, p10 and p20 for 48 h (ii) Percent of scratch closure 48-h post-scratch in fibroblasts co-cultured with CM from the four cell line conditions. n = 3. **c** (i) Number of branches formed by endothelial cells plated on a 3D matrix when co-cultured in various conditions. (ii) Number of meshes formed by endothelial cells plated on a 3D matrix when co-cultured in various conditions. Dotted line represents the average number of (i) branches and (ii) meshes formed in DMEM (control medium). n = 3

We utilized relative absorbance, compared to DMEM, as a proxy for planktonic bacterial growth of *Methicillin resistant S. aureus* (*MRSA*), a method validated in previous work [14, 16, 50]. We found that the CM of primary MDECs lost antimicrobial potency between passages 10 and 15, from approximately 50% reduction in absorbance to 19.5% reduction, relative to the DMEM control and this reduced potency continued until p25 (Fig. 4a). In contrast, CM of immortalized MDECs maintained its antibacterial efficacy of about a 40.5% reduction in absorbance through at least p25 (Fig. 4a). As expected, the antibiotic positive control significantly reduced bacterial growth, illustrated by a 64% reduction in absorbance, as compared to the DMEM control normalized to a 100% absorbance, both shown as dotted lines (Fig. 4a).

In order to evaluate whether the secretomes of other types of bovine stem/progenitor cells affect bacterial growth similarly, CM was collected from bovine mesenchymal stem/stomal cells (MSCs) isolated from adipose tissue (AD), bone marrow (BM) and peripheral blood (PB), and evaluated for antimicrobial effects against *MRSA*. The anti-MRSA effect observed with MDEC CM was not recapitulated using CM of any of these three additional bovine MSCs (Supplemental Fig. 3).

We then compared the pro-migratory potential of CM from primary and immortalized MDECs using a fibroblast scratch assay. The slope of the line indicating the percent of the scratched area that was filled in by migrating fibroblasts over time flattened by 19% when comparing primary MDEC CM from p20 cells to primary MDEC CM from p10 cells, suggesting that p20 CM has less of an effect on fibroblast migration than p10 CM. This change in slope is not observed when comparing the migration of fibroblasts cultured in p20 versus p10 immortalized MDEC CM (Fig. 4b(i)). When comparing the percentage scratch closure by fibroblast migration after 48 h of exposure to MDEC CM, we confirmed that primary MDEC CM collected from p20 cultures did have less of an effect on fibroblast migration than CM collected from p10 cultures, while the effects of immortalized MDEC CM on fibroblast migration did not change from p10 to p 20 (Fig. 4b(ii)).

The angiogenic potential of CM from primary and immortalized MDECs was evaluated using an assay in which bovine lung microvessel endothelial cells (BLMVEC) were plated in 3D culture and their ability to form tubules under various conditions was assessed. The number of branches and the number of meshes were counted as a measure of tubule branching complexity, which has been validated previously as a measure of angiogenic potential [16, 52]. CM from both cell lines at p10 significantly increased the number of endothelial branches and meshes when compared to the DMEM control (Fig. 4c(i) and (ii)). However, when comparing the potency of the CM of both primary and immortalized MDECs at p10 and p20, both the number of branches and the number of meshes significantly decreased indicating the angiogenic potential of primary and immortal MDECs is lost over time in culture (Fig. 4c(i) and (ii)).

To explore whether the insertion of *hTERT* into the MDEC genome may have disrupted the expression of genes commonly associated with angiogenic properties [67], qPCR was performed, comparing p10 primary and immortalized MDECs. No significant difference in expression of vascular endothelial growth factor (*VEGF*), fibroblast growth factor 2 (*FGF2*) or TANK-binding kinase 1 (*TBK1*) was found between the primary and hTERT-immortalized MDECs (Supplemental Fig. 4).

**Fig. 5:**
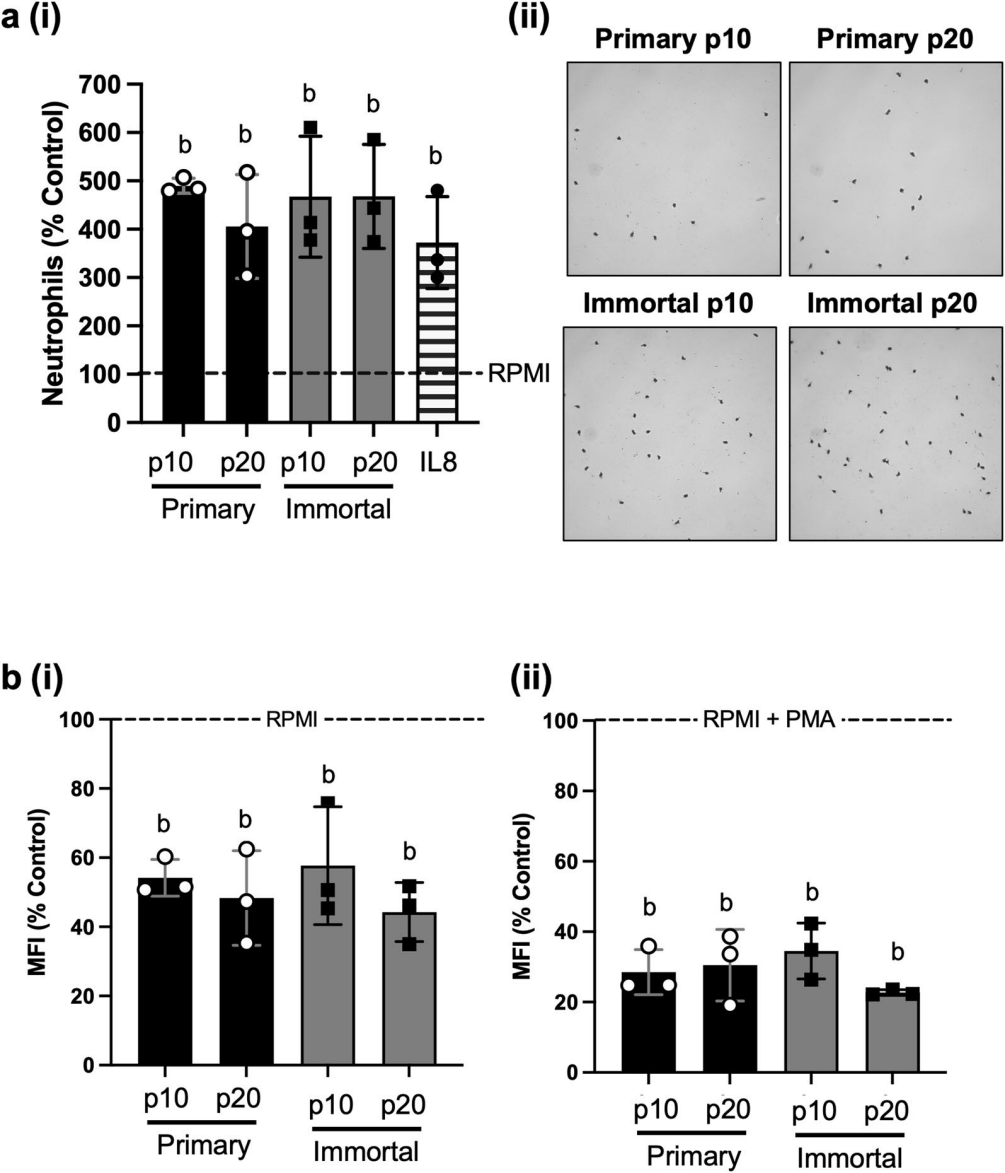
Immortalization and passage of mammosphere derived epithelial cells (MDECs) does not change the effects of MDEC conditioned medium (CM) on neutrophil chemotaxis or reactive oxygen species (ROS) production. **a** (i) Relative chemotaxis as a percentage of the control of neutrophils cultured in primary and immortalized p10 and p20 MDEC CM. A positive control, IL8, is included. (ii) Representative images of neutrophils that migrated in the chemotaxis assay. **b** Relative mean fluorescence intensity (MFI) relative to the control comparing the generation of ROS in neutrophils cultured in primary and immortalized p10 and p20 MDEC CM, respectively. (i) Data from unstimulated neutrophils, (ii) data from neutrophils were stimulated with PMA. n = 3. The same letters on graphs indicate no statistically significant differences between groups

Lastly, we compared the immunomodulatory properties of CM collected from primary and immortalized MDECs at both p10 and p20. Specifically, we evaluated chemotaxis and reactive oxygen species (ROS) production by neutrophils, innate immune cells that exert early defense mechanisms against bacterial infections [68, 69]. We found that MDEC CM, both at p10 and p20, from both primary and immortalized cells, resulted in significantly increased neutrophil chemotaxis when compared to neutrophils that were cultured in RPMI control medium (Fig. 5a). We saw no significant differences in neutrophil chemotaxis between those cultured in any of the MDEC CM groups and those cultured in RPMI supplemented with interleukin 8 (IL8), a chemokine known to promote neutrophil chemotaxis [70] and included as a positive control (Fig. 5a). While a slight decrease in the pro-migratory effects of primary MDEC CM on neutrophils was observed from p10 to p20, it did not reach statistical or biological significance (Fig. 5a). This was not observed in the chemotaxis of neutrophils cultured in p10 and p20 immortalized MDEC CM. When assessing ROS accumulation in neutrophil cultures grown in MDEC CM, we found that all four CM groups tested (i.e. primary and immortalized, and at p10 and p20) significantly reduced the accumulation of ROS, both when neutrophils were unstimulated, and stimulated with phorbol myristate acetate (PMA) (Fig. 5b), relative to those grown in RPMI control medium. A PMA-stimulated condition was added to determine whether MDEC CM affects stimulated as well as resting neutrophils, as neutrophils may be activated during infections [71, 72]. There were no differences in the effects on chemotaxis or ROS production when comparing primary versus immortalized cells, or early passage versus late passage cells. There were no observable trends or differences, biologically nor statistically, in ROS accumulation in neutrophils treated with primary or immortalized p10 or p20 MDEC CM (Fig. 5b).

Collectively, our findings show that the CM of immortalized MDECs maintained antimicrobial and pro-migratory potency over longer subpassages when compared to the CM of primary MDECs, and that the immortalization of MDECs did not restore the loss of angiogenic potential of CM at higher passages. The immunomodulatory effects of MDEC CM on neutrophils did not change regardless of immortalization status or passage.

### Analysis of the proteomic composition of the secretomes from primary and immortalized MDECs at p10 and p20 correlates with their functional properties

**Fig. 6:**
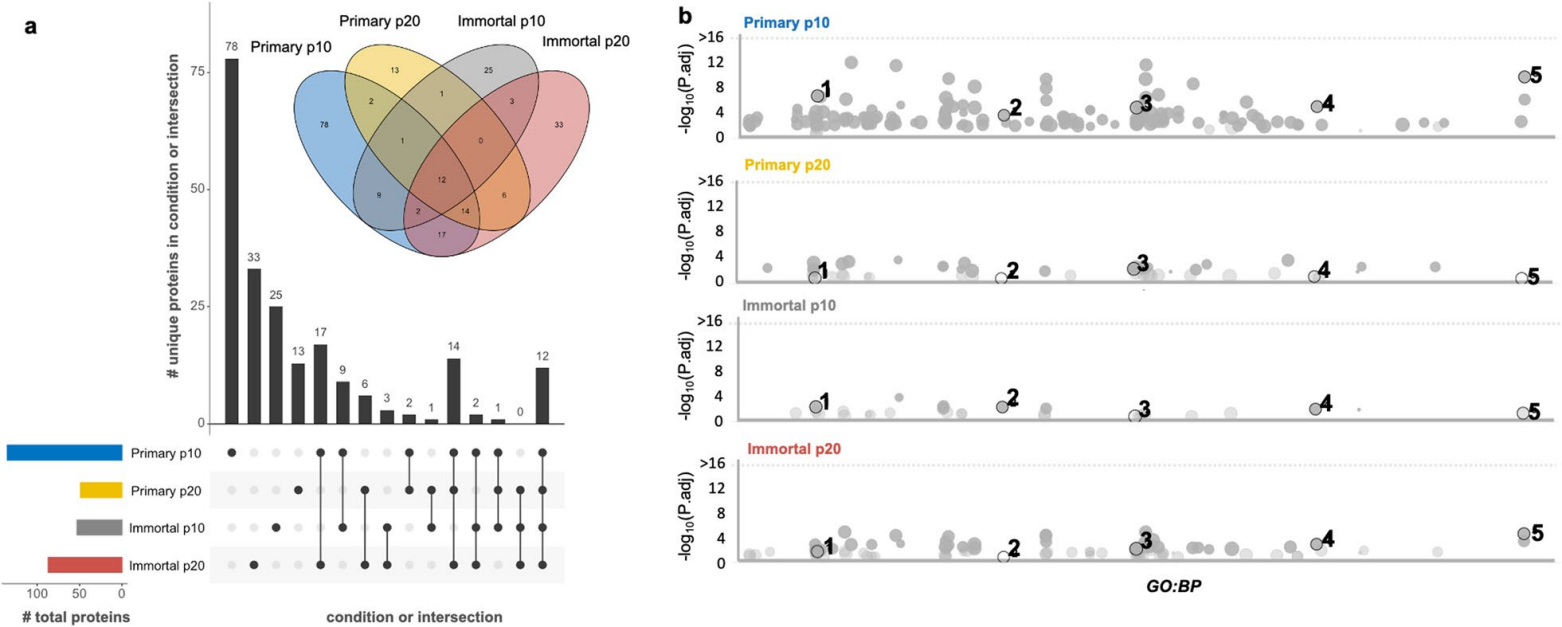
Mass spectrometric analysis reveals differences in proteomic compositions of the conditioned medium (CM) of primary and immortalized p10 and p20 mammosphere derived epithelial cells (MDECs). **a** UpSet plot indicating the total number of proteins in each of the individual test groups (left, bar chart), the total number of unique proteins in each group (top, bar chart, first 4 bars), the total number of shared proteins in each intersection of the conditions (top, bar chart, last 11 bars), as well as a Venn diagram (top) representing all intersections and the number of proteins in each). **b** GO-term enrichment Manhattan plots of GO terms associated with biological processes (BP) enriched in each of the conditions over the log2 of the p-value for each enrichment. Highlighted GO:BPs are described in Table 4 and include: 1—cytoskeleton organization, 2—cell junction organization, 3—animal organ development, 4—supramolecular fiber organization, and 5—regulation of cell motility

We performed mass spectrometry on the CM from primary and immortalized MDECs at both p10 and p20 to look for changes in protein composition that might correlate with the differences in secretome activity observed between the cell lines at different passages. A list of all proteins identified in each CM sample is included in Supplemental Table 1 and proteins with known roles in antimicrobial defense, cell motility, angiogenesis, and immunity [17] are listed in Table 3. The highest number of proteins was detected in the CM from primary MDECs at p10, with most of them (i.e., 78 proteins) being only found in that particular CM (Fig. 6a). The second highest number of total, as well as unique proteins (i.e., 33 proteins), was found in CM from immortalized MDECs at p20, whereas the CM from primary MDECs at p20 and immortalized MDECs at p10 contained the lowest number of identified proteins (i.e., 25 and 13 unique proteins, respectively) (Fig. 6a). In terms of number of proteins shared between 2 CM groups, CM from primary MDECs at p10 and immortalized MDECs at p20 showed the greatest overlap, whereas other intersections in shared proteins were small for most other CM comparisons (Fig. 6a).

**Table 3:** Proteins with properties related to observed functional properties of the MDEC CM across conditions

Protein name	Primary p10	Primary p20	Immortal p10	Immortal p20
*Proteins related to antimicrobial defense and immunity*				
Alpha-1-acid glycoprotein precursor	X	X		X
Alpha-1-antiproteinase precursor	X	X		X
Alpha-2-macroglobulin precursor	X	X		X
Complement C3 preprotein		X		X
*Proteins related to cell motility*				
Annexin A1	X			
Annexin A2	X	X	X	X
Arf-GAP with coiled-coil, ANK repeat and PH domain-containing protein 1			X	
CD44 antigen isoform X6	X			
CLIP-associating protein 1 isoform X3			X	
Fetuin B precursor	X	X		X
Fibronectin isoform X1	X	X	X	X
*Proteins related to angiogenesis*				
CCN family member 1 precursor	X			X
CCN family member 2 precursor	X			
CCN family member 3 precursor	X		X	X

To compile and analyze the data from mass spectrometry across the 4 different CM samples, GO-term enrichment was performed. The number of enriched terms in each CM sample correlated with the overall number of proteins identified in that respective sample (Fig. 6b). Several GO-terms for biological processes shown in Fig. 6b were numbered as follows: 1 – cytoskeleton organization, 2 – cell junction organization, 3 – animal organ development, 4 – supramolecular fiber organization, and 5 – regulation of cell motility. These GO-terms are further explored in Table 4, where their adjusted p values are compared by group. Of note, the GO-terms for cytoskeleton organization and cell junction organization (#1 and 2), both related to angiogenic processes, were found to be diminished in the CM from p20 MDECs, both primary and immortalized (Fig. 6b). This supports our functional findings that primary and immortalized MDEC CM show decreased effects on angiogenic process at p20 than at p10. Similarly, we see a maintained enrichment for animal organ development (#3) in both primary and immortalized p20 groups, a pathway associated with stem/progenitor cell potential (Fig. 6b). Finally, the relative decrease in enrichment of supramolecular fiber organization and cell motility (#4 and 5), both associated with cell migration [73–75], between primary p10 and p20, as opposed to the opposite trend between immortalized p10 and p20, coincides with our findings that immortalized MDEC CM better maintains the migratory effect observed in our functional experiments.

**Table 4:** Differentially expressed GO-terms from the proteomic analysis highlighted in Fig. 6b

Ref	GO ID	GO Term	Primary p10	Immortal p10	Primary p20	Immortal p20
1	GO:0007010	Cytoskeleton organization	7.3 × 10^−8^	1.4 × 10^−2^	7.8 × 10^−1^	8.8 × 10^−2^
2	GO:0034330	Cell junction organization	3.5 × 10^−4^	1.6 × 10^−2^	1	1
3	GO:0048513	Animal organ development	1.2 × 10^−5^	1	1.4 × 10^−2^	2.5 × 10^−2^
4	GO:0097435	Supramolecular fiber organization	8.0 × 10^−6^	3.9 × 10^−2^	4.2 × 10^−1^	3.5 × 10^−3^
5	GO:2000145	Regulation of cell motility	1.7 × 10^−11^	2.4 × 10^−1^	1	3.4 × 10^−5^

## Discussion

Our laboratory has been exploring the secretomes of stem and progenitor cells of various animals, including cows [15–17], horses [14, 24, 49, 76] and chickens [50]. Previous work has shown that the bovine mammary stem/progenitor cell (MDEC) secretome exhibits various antimicrobial, promigratory, immunomodulatory and anticancer properties [13, 16–18]. This study confirmed that the bovine MDEC secretome exhibits antimicrobial properties, and moreover, showed that this is in contrast to the secretomes collected from bovine mesenchymal stromal/stem cells (MSCs) isolated from different tissues sources, i.e. adipose tissue, bone marrow and peripheral blood, where no antimicrobial effects were observed.

By immortalizing bovine primary MDECs we provide a strategy to produce stable, long lived cell cultures with therapeutic potential. The prolonged genomic integrity, phenotypic consistency, and proliferative capacity of immortalized MDECs render them more suitable as a biologic therapy than short-lived primary cell cultures. The improved mammosphere-forming and prolonged colony-forming capacity further solidify immortalized MDECs as a good therapeutic alternative as they maintain their stem cell-like and self-proliferative capacity, which may allow for greater scalability [32, 77, 78]. Secreted factors from late passage immortalized MDECs exert antimicrobial effects on *MRSA*, promote fibroblast migration, stimulate neutrophil chemotaxis, and inhibit neutrophil reactive oxygen species (ROS) production in vitro, suggesting that immortalized primary bovine MDECs may serve as a relevant therapy in vivo*.*

Immortalization of bovine primary cells has been shown to confer properties like extended proliferative capacity, conservation of morphology, and maintenance of expressed protein markers [79]. Here, we observed that the canonical bovine MDEC morphology is retained in immortalized, but not primary, cells at higher passages, an observation similar to human immortalization experiments [80, 81]. In fact, the morphology of late-passage primary MDECs, characterized by larger and rounder more spread-out cells, resembles the commonly observed morphology of cells that lose their proliferative capacity, which includes a “flattened” appearance and an increase in cell size [82]. These findings suggest that the insertion of *hTERT* in bovine MDECs can prolong their lifetime in culture, an outcome which has been validated in other cell types in the bovine model, as well as in multiple other species [79, 83]. Previous work has also shown that bovine primary MDECs lose a significant portion of their mammosphere-forming capacity (MFC) after a second round of subculturing, decreasing from ~ 6% at passage (p) 1 to 2% at p2 [32]. The data in the present study corroborated this finding in the primary bovine MDEC line. In contrast, immortalized bovine MDECs maintained a similar MFC at high passages. Notably, previous work characterizing human mammary epithelial cells isolated through a mammosphere-independent protocol found that immortalizing these cells resulted in a lack of mammosphere formation [84], potentially suggesting that isolating mammary epithelial cells using our previously published mammosphere-formation protocol [17] can maintain mammosphere formation after immortalization. Likewise, we found that immortalized bovine MDECs maintained, and even slightly increased, their colony forming capacity (CFC) [16, 32, 77], similar to what has been previously reported for human keratinocytes immortalized with SV40 [85].

Large animal models, including cattle, have previously been validated as important models for human disease and therapeutics due to multiple factors, including the greater similarity in anatomy and physiology to humans than smaller, more common laboratory models [86–88]. Aside from the obvious differences and similarities in overall body size, there is also greater similarity between bovines and humans in the context of the mammary gland, since humans and cows have fibrous mammary gland stroma whereas the stroma of rodents is heavily adipose-rich [89–91]. Additionally, both pharmaceutical regulatory bodies and international scientific consortia recommend the use of large animal models in the pre-clinical phase of studying advanced therapeutic medicinal products (ATMPs), such as the stem cell secretome [92]. To translate this work from the bovine model to human use, preclinical work including in vitro studies, as described in this study, as well as ex vivo studies, i.e. using mammary gland xenotransplantation studies in mice [93], can be used to study the therapeutic effects and safety profile of the secretome. Moreover, studies such as this combined with other studies from our group [15–17, 29], where the purification and standardization of MDEC CM is explored, can be used as a basis for future safety and efficacy studies, a prerequisite for regulatory compliance in downstream therapeutic applications in humans. Moreover, the utility of the bovine model as a proxy for human health has been demonstrated in cell types other than those from the mammary gland, e.g., in mesenchymal stromal cells, as described in our previous work, and embryonic tissues, as described by others [15, 94–96]. Such findings confirm that the bovine model not only represents a strong choice for therapeutic exploration due to the significant functional effects, but also due to the apparent similarity with human tissues.

In the present study, we found that the bovine MDEC morphology and marker expression aligns with previously described work in human mammary epithelial cells with stem/progenitor characteristics [84]. Indeed, both primary and immortalized bovine MDECs exhibit a cobblestone-like morphology, are plastic adherent, and consistently express stem cell marker *CD49f*, sharing these common features with human mammary epithelial cells. Additionally, a lack of expression of αSMA has been documented in both bovine and human cell lines that otherwise express other markers of the basal myoepithelium [17, 84]. These shared features further validate the cow as a strong alternative model for the study of human stem cell-based therapeutics, as bovine adult stem cells share more common functionally relevant gene expression profiles with human stem cells than compared to murine stem cells [15].

In addition to serving as a model for human health, the economic importance and ubiquity of cows render the bovine model relevant to veterinary medicine as well. Research into common infections of cows, such as mastitis, is imperative to the livelihood of many farmers. Mastitis is an inflammation of the mammary gland, often the result of a bacterial infection, and one of the most common and severe diseases affecting dairy cattle [97, 98]. Annually, 30–70% of all lactating cattle on dairy farms experience one or more cases of clinical or subclinical mastitis, resulting in economic losses that can add up to 11–18% of the gross margin per cow per year [99]. Currently, antibiotics are an indispensable aspect of mastitis treatment due to their ability to inhibit the growth of pathogens in the udder, but also to ensure the welfare of diseased dairy cattle [100–103]. However, the use of antibiotics as the sole treatment for mastitis may not improve long-term milk yields, which is typically decreased after infection [101, 104]. The use of secretome from immortalized MDEC may result in improved outcomes for cows with mastitis by inhibiting bacterial growth and stimulating neutrophil functionality combined with improving tissue regeneration to decrease long-term milk yield losses.

Methicillin-resistant *S. aureus* (*MRSA*) is one common pathogen that evades conventional antibiotic treatments and the host immune response across species, leading to substantial tissue damage [105–108]. In the present study, we found a measurable decrease in *MRSA* growth when co-cultured in secretome collected from early passage primary and immortalized bovine MDECs, as well as late passage immortalized, but not primary, MDECs, showing that immortalizing MDECs prolongs the antibacterial activity of the secretome collected at higher cell passages.

Studies have shown that the migration of fibroblasts to sites of injury plays a critical role in the processes of new tissue synthesis, tissue remodeling and wound healing [19, 20, 109, 110]. The current study, as well as previously published work, has found that the secretome from low-passage bovine mammary stem/progenitor enhances fibroblast migration in vitro [16, 17]. Importantly, the present study showed that the stimulatory effects on fibroblast migration remained when using secretome collected from high passage immortalized, but not primary, MDECs, providing further evidence that immortalization increases the length of time an MDEC line is suitable for the generation of therapeutically relevant secretome.

Certain bacterial pathogens, including *MRSA*, evade neutrophil phagocytosis by influencing their recruitment to the site of infection and/or by replicating inside host cells [111–114]. We previously showed that the bovine MDEC secretome increases neutrophil chemotaxis comparably to the known neutrophil chemoattractant interleukin 8 (IL8) and decreases the production of tissue-damaging ROS by neutrophils [29]. Our current study confirmed these properties of the secretome from both primary and immortalized cells and at both low and high passages. Increasing chemotaxis and decreasing ROS production by neutrophils may reduce the length of active bacterial infections and mitigate the bacterial-mediated tissue damage [111–117], collectively mitigating the long-lasting negative effects often caused by infections. The above findings propose the immortalized MDEC secretome as a strong candidate for the development of novel therapeutic applications, for example based on the potential to modulate the innate immune response in cases of acute bacterial infections, which is currently not addressed with conventional antibiotic treatments. Given that no biological or statistical differences were identified in the immunomodulatory properties of immortalized MDEC CM between p10 and p20, future work could focus on identifying whether there are any subtler differences in neutrophil modulation over time, in order to serve as a better predictor of outcomes in clinical settings.

Previous studies by other groups have found that while *hTERT* can stabilize the expression of certain phenotypes in immortalized cell lines, it may not preserve others, even within the same cell line. For example, in immortalized human endothelial cells, expression of proteins associated with the endothelial phenotype remained constant as a result of immortalization, but expression of other proteins, such as cytokeratin (KRT) 7 and calumenin (CALU), varied despite immortalization [118]. In our work, we found that *hTERT* did not prolong the pro-angiogenic effect of the MDEC CM at p20. To explore whether the insertion of *hTERT* in the genome may have disrupted the expression of pro-angiogenic genes, we compared the expression of vascular endothelial growth factor (*VEGF*), fibroblast growth factor 2 (*FGF2*) and TNK-binding kinase 1 (*TBK*) between p10 primary and *hTERT*-immortalized cells. Our results indicate that the insertion of *hTERT* did not cause a direct off-target effect on genes associated with pro-angiogenic outcomes [67]. Other groups have shown that *hTERT*-induced immortalization of cell lines with known pro-angiogenic effects, such as human pulmonary microvascular endothelial cells, does not modify the angiogenic characteristics of the primary cell line [119]. Additionally, genetic drift within the cell culture could explain the loss of pro-angiogenic capacity over time, as this has been a well-documented phenomenon associated with cultured cell lines [120].

While useful in extending the proliferative capacity of cells in culture, immortalization of cell lines may induce unintended changes in the genome [121]. Such modifications can lead to tumorigenesis and changes in morphology and function [121, 122]. In this study we observed that immortalization with hTERT did not induce immediate chromosomal rearrangement. This has been shown previously in both murine and human cells, where it was found that hTERT did not cause chromosomal rearrangements over multiple subcultures [121, 123–125]. The potential for tumorigenesis is further reduced as the goal of this study was to produce consistently reproducible MDEC secretome as a safer alternative to using cell-based treatments, thus, no cells would be engrafted into host tissues, bypassing concerns commonly associated with cell-based therapeutics [126].

Finally, it is important for therapeutic agents to be readily available for usage. While the process of collecting fresh CM from immortalized MDECs is relatively short, it requires access to a laboratory and can take over a week. A more readily deployable solution would involve the use of long-term stored immortalized MDEC CM. Previous studies have shown that the bioactive secretomes of various cell types can be stored through ultra-freezing (-80 °C) or lyophilization for up to 6 months without significant loss in bioactivity, both in the context of protein- and extracellular vesicle-mediated functional properties [127–131]. Future work should focus on exploring potential storage methods specifically for MDEC CM, evaluating all the observed functional properties after long-term storage by either ultra-freezing or lyophilization. Identifying the durability of the bioactive effects of the MDEC secretome would increase the likelihood of regulatory approval for future therapeutic uses.

## Conclusions

Our findings present immortalized MDECs as a viable therapeutic alternative to the use of primary MDEC lines by providing a secretome that remains therapeutically active at higher cell passages. Additionally, this work promotes the immortalized bovine MDEC model as a viable cross-species approach to the study of cell-free biologic therapies for human use.

## Supplementary Information

Supplement 1: Detailed methods for mass spectrometry assays and analyses

Supplemental Figure 1: Transfection efficiency, selection and verification of immortalized cell lines.Results of flow cytometry from transfection optimization assay with the highest GFP expression.Representative images of mock and GFP-transfected MDECs used for transfection optimization protocols.Survival curve of MDECs at various concentrations of G418 to identify lethal dose. Performed using MTT viability assay.Representative image of transfected MDECs post G418 indicating normal morphology.PCR gel results indicating lack of NeoR/KanR presence in primary MDECs and presence of NeoR/KanR in immortalized MDECs. GAPDH used as loading control

Supplemental Figure 2: Individual results for qPCR data represented in Figure 2. Bar charts representing the mean and standard deviation of each qPCR run for the specific condition and gene. RQ represents relative quantification of mRNA expression relative to GAPDH. n=3. Letters represent statistical significance groups

Supplemental Figure 3: Antimicrobial properties of bovine adipose tissue-derived, bone marrow-derived, and peripheral blood-derivedmesenchymal stromal cellssecretomes, collected as conditioned mediumagainst Methicillin resistant S. aureus. Relative absorbance of *MRSA* when co-cultured with CM from bovine AD-, BM- and PB-MSCs at p6. Upper dotted line represents the absorbance of *MRSA* cultured in unmodified DMEM, and the ABX bar represents a positive control of medium with antibiotics. Different letters on graphs indicate statistically significant differences between the groups and the DMEM control. n=3.

Supplemental Figure 4: Comparison of qPCR relative quantification of genes associated with angiogenesis in p10 primary versus immortalized MDECs. Bar charts representing the mean and standard deviation for qPCR assays performed on p10 primary and immortalized MDECs comparing the expression of three genes associated with angiogenesis. RQ represents relative quantification of mRNA expression relative to *GAPDH*. n=3. No significant differencewas detected across all three genes.

Supplemental Table 1: List of all identified proteins in conditioned media of primary and immortalized MDECs at passages 10 and 20. Excel file containing tabs for each of the conditionslisting all proteins identified in the mass spectrometric analysis of their conditioned media

## Data Availability

The dataset generated by this study is available in Supplemental Table S1 of this manuscript. Any additional data or materials will be provided by the corresponding author on reasonable request.

## Abbreviations

bFGF: Basic-fibroblast growth factor
BLMVEC: Bovine lung microvascular endothelial cells
CFC: Colony forming capacity
CFU: Colony forming units
CM: Conditioned medium
DMEM: Dulbecco’s Modified Eagle Medium
EDTA: Ethylenediaminetetraacetic acid
EGF: Epithelial growth factor
EpSC: Epithelial stem cell
EtOH: Ethanol
h: Hour
hEST2: Human EST 2
hTERT: Human telomerase reverse transcriptase
IF: Immunofluorescent
MDECs: Mammosphere-derived epithelial cells
MFC: Mammosphere-forming capacity
min: Minute
MRSA: Methicillin resistant *Staphylococcus aureus*
MTT: 3-(4,5-Dimethylthiazol-2-yl)-2,5-diphenyltetrazolium bromide
p: Passage
PBS: Phosphate buffered saline
PDT: Population doubling time
ROS: Reactive oxygen species
*S. aureus*: *Staphylococcus aureus*
sec: Seconds
