# Supplementary material for: Immortalized mammosphere-derived epithelial cells retain a bioactive secretome with antimicrobial, regenerative, and immunomodulatory properties
Source: Stem Cell Res Ther. 2024 Nov 14;15:429. doi: 10.1186/s13287-024-04019-1 (PMC11566417; doi:10.1186/s13287-024-04019-1)
Supplement: Supplementary file 1 — Supplement 1: Detailed methods for mass spectrometry assays and analyses [file 13287_2024_4019_MOESM1_ESM.docx]

Danev et al., “Immortalized mammosphere-derived epithelial cells retain a bioactive secretome with antimicrobial, regenerative, and immunomodulatory properties”

**Supplementary Methods 1:** **Mass spectrometry methods**

CM in phenol red-free and PBS-free DMEM was collected, and 1% Halt protease inhibitor cocktail was added to each sample. Amicon Ultra-15 filter units with 30 kDa filters were used to fractionate CM for 20 min at 4,000 x g at RT. All volumes were brought up to 5 mL using phenol red-free and PBS-free DMEM. Samples were lyophilized overnight and then stored at -80°C for further processing. Upon collection of all samples, the lyophilized samples were solubilized with 500 µL of 6M guanidinium chloride (GdnHCl), 50 mM Sodium Phosphate, pH 7.0, 10mM dithiothreitol (DTT). Approximately 6-10 µg of protein were removed and solubilization buffer to a final volume of 50 µL was added. Samples were incubated for 1 h at 60 ºC to complete the reduction in DTT, after which they were alkylated with 5.0 µL of 0.6 M iodoacetamide to a final concentration of 54 mM, incubated for 45 min in the dark at RT and then quenched by 5.0 µL of 0.5 M DTT to a final concentration of 42 mM. Samples were diluted with 300 µL o f 50 mM ammonium bicarbonate for a final concentration of GdnHCl of less than 1M, followed by digestion with 7.4 μL of 0.5 µg/µL trypsin to a final concentration of 10 ng/µL (3.7 µg Trypsin-Promega). All samples were incubated overnight at 37 °C. The reaction was quenched with 3 µ L of 100% formaldehyde (FA), then dried to dryness in a speed vacuum.

The dried digests were reconstituted in 0.5 mL 0.1% trifluoroacetic acid (TFA) in water (v/v) and prepped by solid phase extraction (SPE) using SOLA HRP (Thermo Fisher) 10 mg x 1 mL cartridges. The cartridges were conditioned with 0.5 mL methanol and equilibrated with 0.5 mL 0.1% TFA in water (v/v). Samples were loaded at ~2 seconds/drop and washed with 0.5 mL 0.5% FA in water (v/v) followed by 0.5 mL 0.1% FA in water (v/v). The cartridges were briefly blown dry and eluted with 0.5 mL 30% ACN in water (v/v) into a clean 1.7 mL low binding microcentrifuge tube and dried in a SpeedVac. Samples were stored at -20 °C until reconstitution for analysis.

The analysis was carried out using an Orbitrap FusionTM TribridTM (Thermo Fisher) mass spectrometer equipped with a nanospray Flex Ion Source and coupled with a Dionex UltiMate 3000 RSLCnano system (Thermo Fisher). The peptide samples (10 μL) were injected onto a PepMap C-18 RP viper trapping column (5 µm, 100 µm i.d x 20 mm) at 20 µL/min flow rate for rapid sample loading and then separated on a PepMap C-18 RP nano column (2 µm, 75 µm x 25 cm) at 35 °C. The tryptic peptides were eluted in a 90-min gradient of 5% to 35% ACN in 0.1% formic acid at 300 nL/min, followed by an 8-min ramping to 90% ACN-0.1% FA and an 8-min hold at 90% ACN-0.1% FA. The column was re-equilibrated with 0.1% FA for 25 min prior to the next run. The Orbitrap Fusion was operated in positive ion mode with spray voltage set at 1.2 kV and source temperature at 275°C. External calibration for FT, IT and quadrupole mass analyzers were performed. In data-dependent acquisition (DDA) analysis, the instrument was operated using FT mass analyzer in MS scan to select precursor ions followed by 3 second “Top Speed” data-dependent CID ion trap MS/MS scans at 1.6 m/z quadrupole isolation for precursor peptides with multiple charged ions above a threshold ion count of 10,000 and normalized collision energy of 30%. MS survey scans at a resolving power of 120,000 (fwhm at m/z 200), for the mass range of m/z 300-1600. Dynamic exclusion parameters were set at 50 s of exclusion duration with ±10 ppm exclusion mass width. All data were acquired under Xcalibur 4.4 operation software (Thermo Fisher).

The DDA raw files with MS and MS/MS were subjected to database searches using Proteome Discoverer (PD) 2.4 software (Thermo-Fisher) with the Sequest HT algorithm. The PD 2.4 processing workflow containing an additional node of Minora Feature Detector for precursor ion-based quantification was used for protein identification and relative quantitation of identified peptides and their modified forms. The database search was conducted against *Bos Taurus* NCBI database which contains 47394 sequences. The peptide precursor tolerance was set to 10 ppm and fragment ion tolerance was set to 0.6 Da. Oxidation of M, deamidation of N and Q were specified as dynamic modifications of amino acid residues; protein N-terminal acetylation, M-loss and M-loss plus acetylation were set as a variable modification; carbamidomethyl C was specified as a static modification. Only high confidence peptides defined by Sequest HT with a 1% FDR by Percolator were considered for confident peptide identification.
