# Supplementary figures and images for: Immortalized mammosphere-derived epithelial cells retain a bioactive secretome with antimicrobial, regenerative, and immunomodulatory properties
Source: Stem Cell Res Ther. 2024 Nov 14;15:429. doi: 10.1186/s13287-024-04019-1 (PMC11566417; doi:10.1186/s13287-024-04019-1)

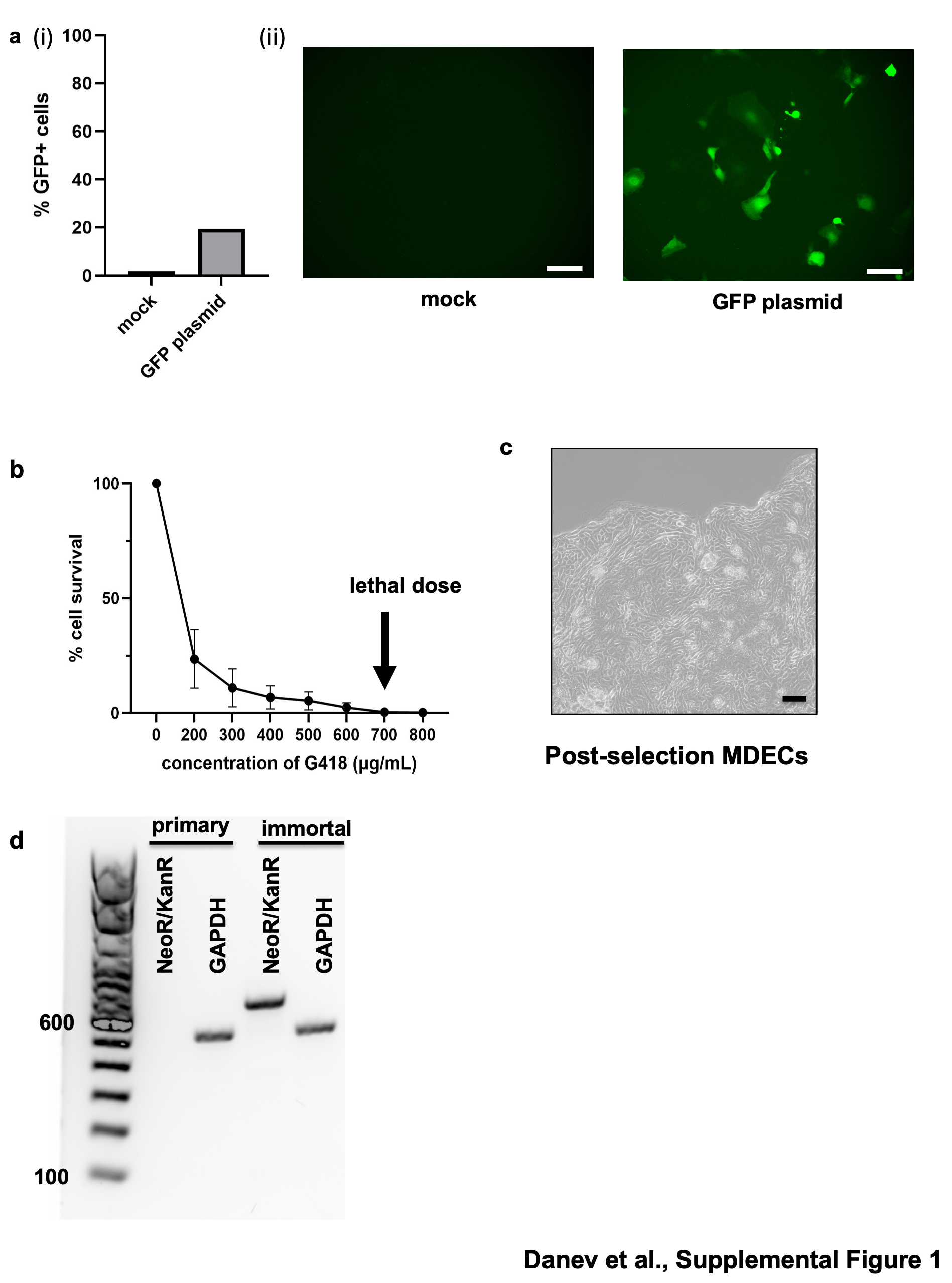

Supplement: Supplementary file 2 — Supplemental Figure 1: Transfection efficiency, selection and verification of immortalized cell lines.Results of flow cytometry from transfection optimization assay with the highest GFP expression.Representative images of mock and GFP-transfected MDECs used for transfection optimization protocols.Survival curve of MDECs at various concentrations of G418 to identify lethal dose. Performed using MTT viability assay.Representative image of transfected MDECs post G418 indicating normal morphology.PCR gel results indicating lack of NeoR/KanR presence in primary MDECs and presence of NeoR/KanR in immortalized MDECs. GAPDH used as loading control [file 13287_2024_4019_MOESM2_ESM.tiff]

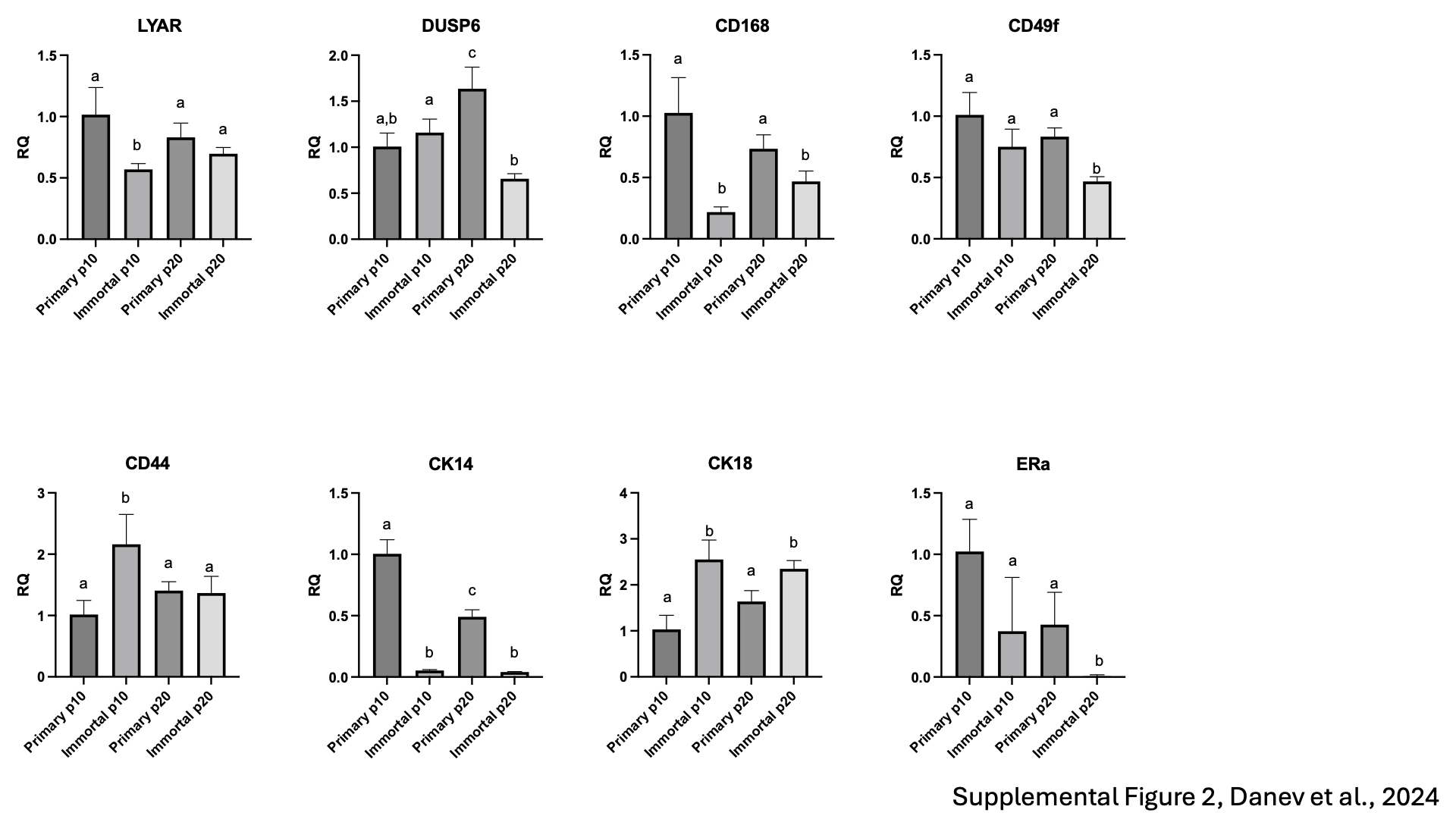

Supplement: Supplementary file 3 — Supplemental Figure 2: Individual results for qPCR data represented in Figure 2. Bar charts representing the mean and standard deviation of each qPCR run for the specific condition and gene. RQ represents relative quantification of mRNA expression relative to GAPDH. n=3. Letters represent statistical significance groups [file 13287_2024_4019_MOESM3_ESM.tiff]

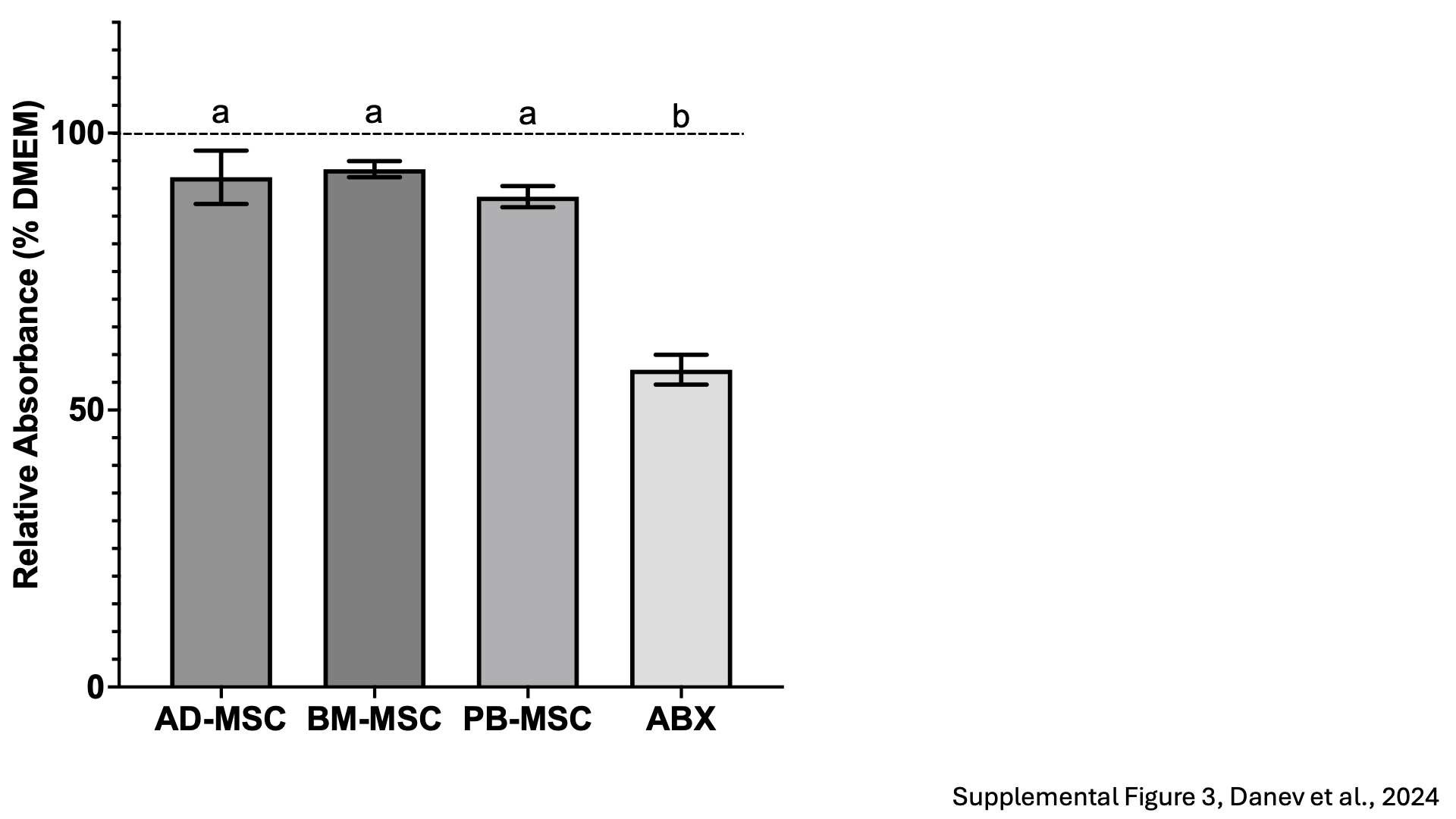

Supplement: Supplementary file 4 — Supplemental Figure 3: Antimicrobial properties of bovine adipose tissue-derived, bone marrow-derived, and peripheral blood-derivedmesenchymal stromal cellssecretomes, collected as conditioned mediumagainst Methicillin resistant S. aureus. Relative absorbance of MRSA when co-cultured with CM from bovine AD-, BM- and PB-MSCs at p6. Upper dotted line represents the absorbance of MRSA cultured in unmodified DMEM, and the ABX bar represents a positive control of medium with antibiotics. Different letters on graphs indicate statistically significant differences between the groups and the DMEM control. n=3. [file 13287_2024_4019_MOESM4_ESM.tiff]

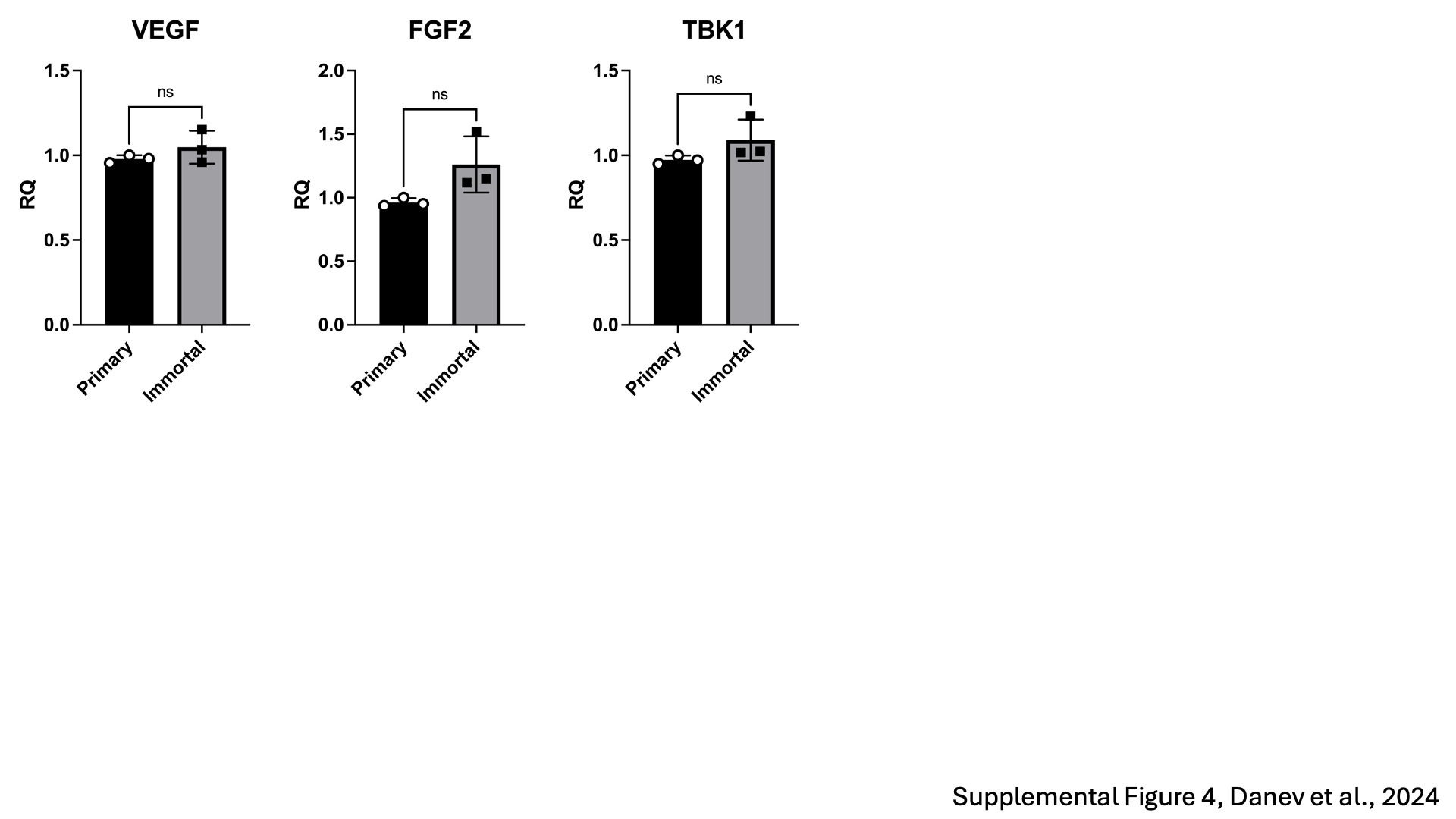

Supplement: Supplementary file 5 — Supplemental Figure 4: Comparison of qPCR relative quantification of genes associated with angiogenesis in p10 primary versus immortalized MDECs. Bar charts representing the mean and standard deviation for qPCR assays performed on p10 primary and immortalized MDECs comparing the expression of three genes associated with angiogenesis. RQ represents relative quantification of mRNA expression relative to GAPDH. n=3. No significant differencewas detected across all three genes. [file 13287_2024_4019_MOESM5_ESM.tiff]
